# Biophysical characterization of *α*-glucan nanoparticles encapsulating feruloylated soy glycerides (FSG)

**DOI:** 10.1016/j.btre.2023.e00817

**Published:** 2023-11-02

**Authors:** Kervin O. Evans, David L. Compton, Christopher D. Skory, Michael Appell

**Affiliations:** a USDA, Agricultural Research Service, National Center of Agricultural Utilization Research, Renewable Product Technology Research Unit, 1815 N. University Street, Peoria, IL 61604, United States of America; b Mycotoxin Prevention and Applied Microbiology Research, 1815 N. University Street, Peoria, IL 61604, United States of America

**Keywords:** α-glucan, Feruloyl soy glycerides, Nanoparticles, Encapsulation

## Abstract

Water insoluble *α*-glucans that were enzymatically synthesized using glucansucrase that was cloned from *Leuconostoc mesenteroides* NRRL B-1118 were previously shown to form nanoparticles via high pressure homogenization. These *α*-glucan nanoparticles were previously shown capable of encapsulating a small hydrophobic molecule. This work demonstrates that the same *α*-glucan can be formed into nanoparticles that encapsulate feruloylated soy glycerides from modified soybean oil, a product of interest to the cosmetic and skin care industries because of the UV absorbance and antioxidant properties of the feruloyl moiety. It is demonstrated that the feruloylated soy glyceride/*α*-glucan nanoparticles have distinct size, zeta potential and thermal profiles from that of nanoparticles made from *α*-glucan alone or feruloylated soy glyceride alone. Thermal analysis also demonstrates the release of feruloylated soy glycerides from the *α*-glucan nanoparticles.

## Highlights

• Alpha-glucan polysaccharides encapsulates feruloyl soy glycerides (FSG) to form novel nanoparticles via high-pressure homogenization.
• Alpha-glucan-FSG nanoparticles are 1.7- and 1.1-times larger than alpha-glucan nanoparticles and FSG nanoparticles, respectively.
• Thermal analysis demonstrates that alpha-glucan-FSG nanoparticles are stable up to about 66 °C.

## Graphical abstract

**Figure:**
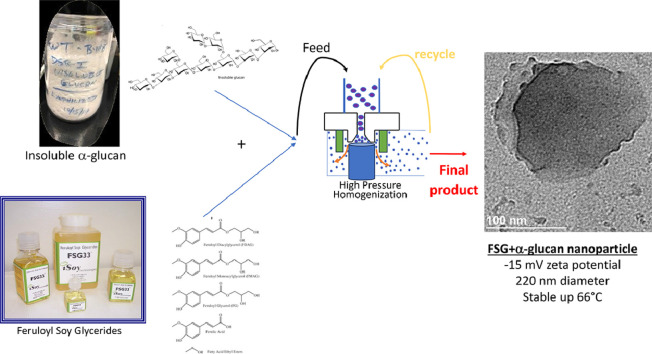

## 1 Introduction

Nanoparticles are defined as materials whose sizes are in the submicron (< 10^−6^ m) range. This covers material from 1 nm (10^−9^) to several hundred nm in size. Particles within this size range have properties that make them of interest in various research fields such as electronics [1], optics [2], drug delivery [3], [4], [5], veterinary medicine [6] and food/nutrition [2]. Nanoparticles are made of various materials to accommodate these different applications. Nanoparticles consisting of metal colloids, for instance, are used to immobilize proteins to maintain favorable orientations on electrodes [1]. Nanoparticles made of dendritic materials are used as direct transporters of pharmacologicals to cells [7]. Metallic colloids [8], [9], [10] and dendrimers [11], [12], [13], however, have been shown to have cell disrupting capabilities when they are below 100 nm in diameter, especially when they are between 4 and 20 nm in diameter [8], [9], [10], [11], [12], [13]. This cell disrupting capability presents a potential biological toxicity issue. There is also a great concern that nanoparticles made from metals, especially heavy metals, have long half-lives in the environment and may create an environmental hazard. There has, therefore, been a push for more “natural”, environmentally friendly material from which to make nanoparticles.

Polysaccharides have been of particular interest for synthesis of nanoparticles because they are abundant in nature or readily produced by enzymatic and/or microbial synthesis [14]. Additionally, polysaccharides are biodegradable and inherently materials composed of polysaccharides are likely to maintain biodegradability.

There are numerous examples where polysaccharides have been used for production of nanoparticles. Chitosan, a linear polysaccharide composed of D-glucosamine and acetyl-D-glucosamine for example, is found abundantly through extraction from crustacean shells like lobsters, crabs, and shrimp. And, chitosan has been used to form nanoparticles for encapsulating essential oils [3]. Alginate, a polysaccharide made of a linear arrangement of β-D-mannuronic acid and α-L-guluronic acid, which is readily extracted from the cell walls of brown algae, has been used to form nanocapsules loaded with turmeric oil [15]. Cellulose, an insoluble polysaccharide readily found in plant cell walls and composed of linear chains of glucose, had been combined with chitosan to encapsulate citronella [16]. Pectin, a polysaccharide composed of at least 17 different monosaccharides and readily found in root vegetables like sugar beets, fruit peel like mango and banana, and nuts like pistachio has been combined with electrically charge polymers to create nanoparticles for encapsulating decosahexanoic acid, D-limonene, orange peel oil, and eugenol oil [17] (and references therein).

Dextran-based polysaccharides are of particular interest for nanoparticle formation. Dextrans are synthesized by extracellular glucansucrase produced by lactic acid bacteria that catalyze the transfer of glucosyl residues from sucrose to the dextran polymer with the subsequent release of fructose. Dextrans consist primarily of a linear *α*(1→6)-linked D-glucopyranosyl backbone modified by minor branching with *α*(1→2), *α*(1→3), and *α*(1→4)-linkages. It is the *α*(1→6) linkage that makes dextrans water-soluble. A shift in linkages from linear *α*(1→6) chains to more *α*(1→3) branching results in the dextrans becoming water-insoluble.

We, in previous work, have demonstrated that a cloned glucansucrase from *Leuconostoc mesenteroides* NRRL B-1118 produced water-insoluble *α*-D-glucan with approximately equal amounts of *α*(1 → 3)-linked and *α*(1 → 6)-linked D-glucopyranosyl units and a low degree of branching [18]. Unlike dextran, these glucans are water insoluble due to the presence of long sequences of *α*(1 → 3)-linked D-glucopyranosyl units. We further showed that high-pressure homogenization of the water-insoluble *α*-glucan could be utilized to form nanoparticles which were capable of encapsulating small hydrophobic bioactives [19,20]. We also demonstrated that water-insoluble glucans nanoparticles combined with cellulose can form films [20].

There has been some interest expressed for films that have ultraviolet (UV)-protection coating. Feruloylated soybean glycerides (FSG) have been shown to have UV-absorbing properties [21] and the ability to protect bioactives from UV degradation [22]. Previous work has shown also that individual components of FSG can be readily encapsulated within lipid nanoparticles [23,24] or starch-based nanoparticles [25]. The current work presented here was conducted to demonstrate the ability of FSG to be encapsulated within α-glucan nanoparticles as a larger hydrophobic bioactive that has ultraviolet (UV)-absorbing and free radical savaging capability [26] (FSG used in this work was not isolated for its components). We characterized physical differences between the nanoparticles containing no FSG and those encapsulating FSG. We characterized the nanoparticles using size, zeta potential, cryo-transmission electron microscopy (TEM), and thermal measurements.

## 2 Materials and methods

### 2.1 Materials

A model C5 high-pressure homogenizer was purchased from Avestin, Inc. (Ottawa, ON, Canada). Purified water used in this work was obtained from a Barnstead Nanopure Diamond UV ultra-purification water system and used at 18.2 MW-cm resistivity.

### 2.2 α-glucan synthesis

α-Glucan was synthesized as previously described [18]. Briefly, synthesis of the *α*-glucans from sucrose was conducted using a glucansucrase that was cloned from *L. mesenteroides* NRRL B-1118 (ATCC 8293). Insoluble material was collected by centrifugation and washed several times with water followed by. Freeze-drying and storage.

### 2.3 Feruloylated soy glycerides (FSG) synthesis

FSG was synthesized as described earlier [26]. Briefly, soybean oil was mixed with ethyl ferulate at 65–75 ºC to form a completely melted and miscible solution. Novozym 435 was added to the miscible solution, resulting in transesterification of ethyl ferulate to soybean oil. Final FSG products were confirmed by HPLC-MS analysis and ^1^H and ^13^C NMR spectral analysis.

### 2.4 Nanoparticle formation

Nanoparticles were formed as previously described [20] with some modification. Dried *α*-glucan (100 mg) was mixed with FSG (200 mg) at 1:2 w/w ratio in a 50 ml conical vial. The vial was gradually rotated back and forth to ensure maximum coating of the dry a-glucan by FSG. Nanopure water was added (40 mL) to give final concentrations of 0.25 % and 0.5 % w/v for the *α*-glucan and FSG, respectively. The solution was stirred overnight and homogenized at 70 MPa and 60 passes, resulting in a clear solution.

### 2.5 Size and zeta potential measurements

A Zetasizer Nano ZPS (Malvern, UK) with a 633 nm/4 mW He-Ne red laser was used to characterize size and zeta potential of the nanoparticles; detection was done at an 173º angle. Each measurement was conducted at 25 ºC where each measurement lasted up to 13 runs, 20 s each run. Nanoparticle reported sizes and zeta potentials are the result of at least experimental runs averaged together.

### 2.6 Cryo-transmission electron microscopy (cryo-TEM)

Nanoparticle samples were prepared as above. Samples were frozen in a dry ice/liquid nitrogen slurry using a Vitrobot System (ThermoFisher Scientific, St. Louis, MO). Samples were visualized using a JEOL 2100 cryo-TEM (JEOL Ltd., Tokyo, Japan).

### 2.7 Nano differential scanning calorimetry (nDSC)

A Nano DSC (TA Instruments, Wood Dale, IL) was used to determine the degradation temperature of nanoparticles. Nanoparticles were created as described above. Nanoparticle samples were scanned from 5 ºC to 125 ºC at 1 ºC/min under 3 atm (304 kPa) pressure. Multiple scans (at least two) were conducted to detect any reversibility.

### 2.8 In silico analysis

Structures of FSG components (ferulic acid, ethyl ferulate, FG, F_2_G, FMAG, and FDOG) were modeled using the semi-empirical PM3 forcefield as implemented in Hyperchem.

### 2.9 Absorbance spectrum

Absorbance spectra were recorded on a Shimadzu UV–Vis spectrophotometer (model UV-1280) over the range of 200 nm–400 nm for each sample. The spectrophotometer was equipped with a 5 nm spectral bandwidith. Backgrounds of solvent only were subtracted pre-scan. Aliquots of 1 mL were used as samples and scans were repeated in triplicate.

### 2.10 Encapsulation rate

Encapsulation rate for FSG within *α* glucan nanoparticles was determined by the following formula: $Encapsulation\;Rate\%=\frac{(Abs\;@\;323\;nm\;ofFSG/\alpha -glucan\;in\;water)*dilution\;factor1}{(Abs\;@\;323\;nm\;of\;FSG\;in\;water)*dilution\;factor2}*100$, where dilution factor 1 and dilution factor 2 are 10 (1of 1 mL of FSG/*α* glucan nanoparticles in water placed into 9 mL of water, and 1 mL of homogenized FSG in water in 9 mL of water).

## 3 Results and discussion

### 3.1 Impact of FSG incorporation on size and zeta potential of *α*-glucan nanoparticles

**Fig. 1:**
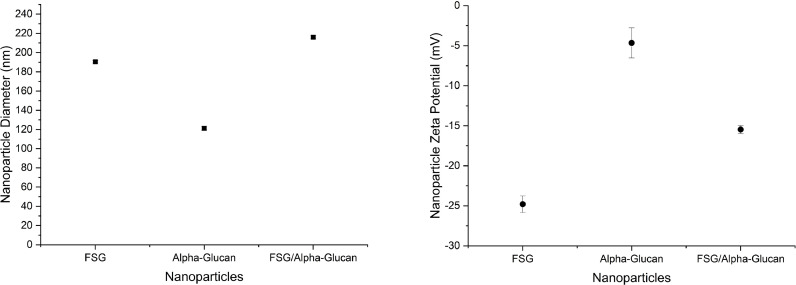
a: Average particle size of FSG, *α*-glucan, and FSG-*α* glucan nanoparticles. Standard deviation was calculated using $\sqrt{\frac{\sum {|x-\bar{x}|}^{2}}{n}}$ where *n* = 9. b: Average zeta potential of FSG, α-glucan, and FSG-*α* glucan nanoparticles. Standard deviation was calculated using $\sqrt{\frac{\sum {|x-\bar{x}|}^{2}}{n}}$ where *n* = 9.

We previously demonstrated that water insoluble *α*-glucans subjected to high-pressure homogenization would form *α*-glucan nanoparticles that were roughly 120 nm in diameter and water soluble [19]. These *α* glucan nanoparticles also were determined to have a zeta potential of approximately ∼ −5 mV. FSG, for comparison, formed nanoparticles with a diameter of approximately 190 nm (Fig. 1a) and a zeta potential of nearly −25 mV (Fig. 1b). This zeta potential value for FSG nanoparticles is well within the range of nanoparticles predicted to not aggregate [27]. Inclusion of FSG with *α* glucan prior to high-pressure homogenization resulted in nanoparticles of approximately 216 nm in diameter and −15 mV. The polydispersity index indicates that nanoparticle heterogeneity remained nearly the same (0.259 ± 0.02, 0.244 ± 0.009, and 0.239 ± 0.008) for FSG, *α* glucan and FSG/ *α* glucan nanoparticles, respectively. This indicates that particle size distribution was not significantly impacted by the incorporation of FSG.

The negative zeta potential demonstrated by FSG nanoparticles is likely due to the maximal amount of feruloyl moieties being exposed on the surface. The charge responsible for this negative zeta potential is presumably due to the deprotonated hydroxy group on the feruloyl moiety that was found at the water interphase in liposomes [24]. It would be expected that the zeta potential of *α* glucan nanoparticles incorporating FSG would lie between −5 mV and −25 mV which are that of individual *α* glucan nanoparticles [19] and FSG nanoparticles, respectively. The FSG-*α* glucan nanoparticles exhibited a zeta potential which was about in the middle of the values for individual *α* glucan nanoparticles and FSG nanoparticles. This suggests that half of the FSG exposed to the outer surface of FSG nanoparticles is now exposed to the outer surface of *α* glucan nanoparticles when incorporated together. Otherwise, it would be expected that the zeta potential of FSG-*α* glucan nanoparticles should be closer to the value exhibited by FSG nanoparticles only if most of the FSG was on the surface-water interface, or closer to the zeta potential of *α* glucan nanoparticles if most of the FSG was incorporated internally of *α* glucan nanoparticles.

Our previous work determined that the *α* glucan nanoparticles contain an internal hydrophobic region [19]. It would be expected that the hydrophobic region of FSG molecules would extend within the hydrophobic region of *α* glucan nanoparticle. The hydrophilic region of FSG molecules which encompasses the feruloyl moiety, however, would most likely to reside near any water interphase as the feruloyl moiety in feruloyl dioleoylglyerol was demonstrated to do so in phospholipid nanoparticles [24]. It, thus, stands to reason that there may be pockets of entrapped water inside of the FSG-*α* glucan nanoparticles to energetically accommodate any internally exposed feruloyl moieties. However, these components vary considerably in their properties (see Table 1). As seen in Table 1, the molecular masses vary from 194 to 880 amu. The volumes vary from 593 to 2590 Å^3^). The lipophilicity index is between 0.83 and 12.92. The hydration energy varies from −3.28 kcal mol^−2^ to −24.72 kcal mol^−2^. It is noteworthy that FMAG (feruloyl monoacylglycerol) and FDOG (feruloyl dioleoylglycerol) have the highest logP values, indicating that these desired value-added bioproducts bind more favorably to the hydrophobic regions of the polysaccharide nanoparticles. In contrast, the reagents and side products are more hydrophilic and less likely to bind the hydrophobic regions of the nanoparticles.

**Table 1:** Parameters of FSG components.

	Mass (amu)	Volume (Å^3^)	logP	Hydration energy (kcal mol^−2^)
FA	194.19	593.08	1.62	−14.31
EF	222.24	714.08	1.99	−9.62
FG	268.27	784.05	0.83	−19.74
F_2_G	444.44	1257.27	2.74	−24.72
FMAG	518.69	1721.50	6.88	−11.44
FDOG	769.11	2579.29	12.92	−3.28

• FA – ferulic acid; EF – ethyl ferulate; FG – feruloyl glycerol; F_2_G – 1,3-diferuloyl glycerol; FMAG – feruloyl monoacylglycerol; FDOG – feruloyl dioleoylglyerol.

### 3.2 Cryo-TEM measurements

**Fig. 2:**
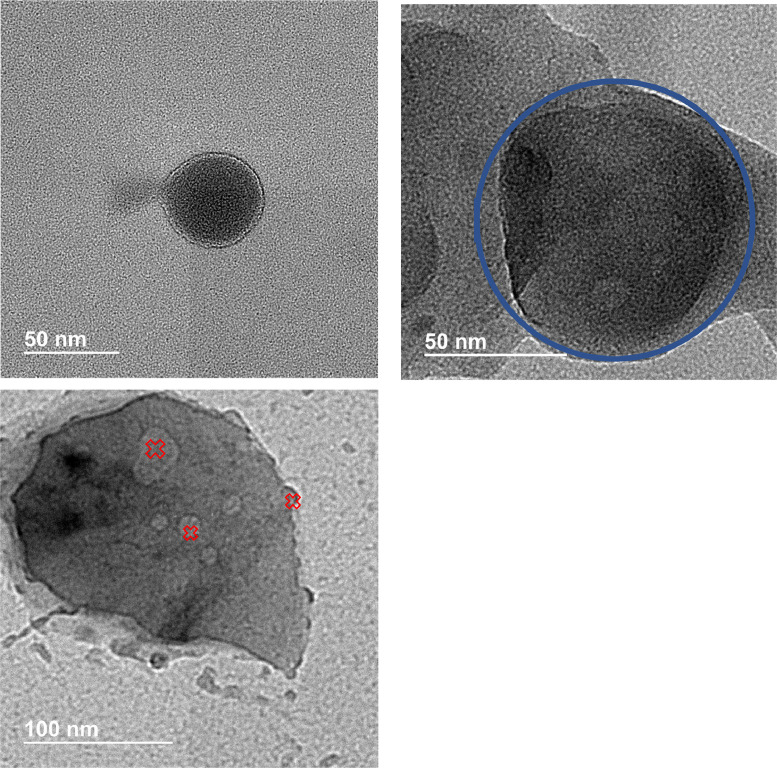
a: Cryo-TEM image of FSG nanoparticle liposome rupturing. Scale bar is 50 nm. b: Cryo-TEM image of *α*-glucan nanoparticle highlighted by blue circle with a scale bar of 50 nm. c: Cryo-TEM image of FSG-*α* glucan nanoparticle. *Red hollow x* highlights possible localized FSG on the surface of an *α* glucan nanoparticle. Scale bar is 100 nm.

Cryo-TEM was undertaken to determine structural features of the FSG-*α* glucan nanoparticles. Fig. 2a shows a typical FSG nanoparticle which was roughly 50 nm in diameter under the cryogenic conditions during the measurement. It is noticeable that high-pressure homogenized FSG nanoparticles were uniformly spherical in shape and appear to have a thin layer of material surrounding water; Fig. 2a shows one FSG nanoparticle as it ruptured, expelling its internal liquid contents. This spherical shape and thin layer of material is consistent with cryo-TEM images of liposomes [28] which exhibit a unilamellar bilayer of lipids encapsulating an internal liquid core. We are interpreting the image of the FSG nanoparticle as having a unilamellar bilayer of FSG encapsulating water.

Fig. 2b shows an *α* glucan nanoparticle as having a non-uniform spherical shape under cryogenic conditions. This is still similar in shape to the spherical structures exhibited when dried for SEM measurements and similar in size to those in water as determined earlier [19].

Fig. 2c shows an FSG-*α* glucan nanoparticle which took on more of a teardrop shape under cryogenic conditions. It is also noticeable that the edges are more rugged than what was seen for the *α* glucan nanoparticles in Fig. 2b. There are areas that also appear more globular in nature (marked by *red hollow x* in Fig. 2c). We interpret these as regions of FSG on the surface. We, however, cannot rule out that FSG mostly coats the surface of *α*-glucan nanoparticles as the dark layer surrounding the teardrop nanoparticle in Fig. 2c may be.

### 3.3 Nano DSC

Previous work demonstrated that *α*-glucan exhibited a thermal profile in water that suggested structural changes occurring around 70–80 ºC [19]. We furthered analyzed the *α*-glucan nanoparticles, FSG nanoparticles and FSG-*α* glucan nanoparticles using the nano DSC because it can accommodate pressure issues associated with boiling obtained at temperatures at or above 100 ºC.

**Fig. 3:**
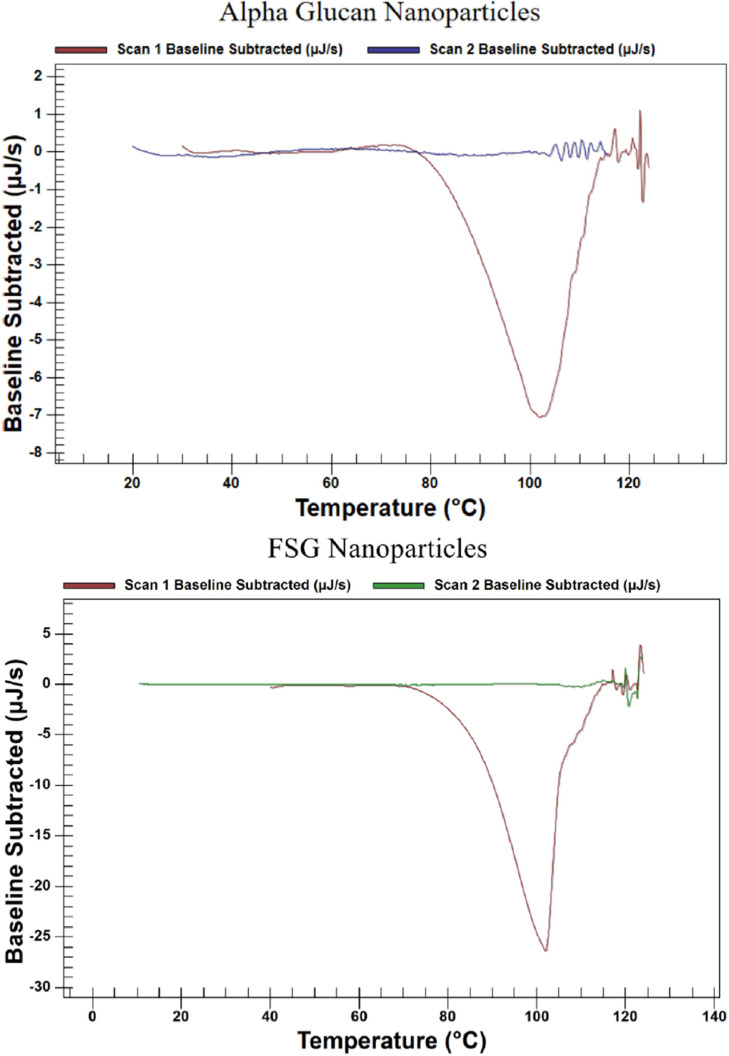

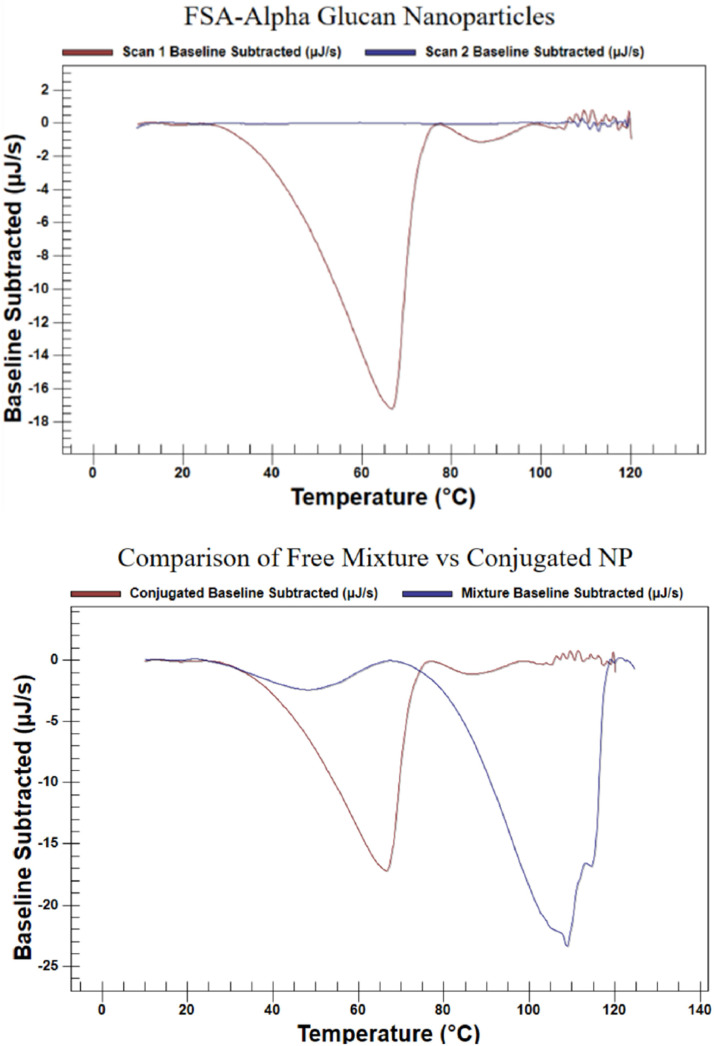
a: Nano DSC thermograph of *α*-glucan nanoparticles in water. The red line is the initial scan; the blue line is the subsequent scan of the same sample after cooling the sample back to 20 ºC. b: Nano DSC thermograph of FSG nanoparticles in water. The red line is the initial scan; the blue line is the subsequent scan of the same sample after cooling the sample back to 20 ºC. c: Nano DSC thermograph of FSG-*α* glucan nanoparticles in water. The red line is the initial scan; subsequtent scan indicating complete sample degradation is represented by the blue line. d: Nano DSC thermograph of 1:1 mixture of free *α*-glucan nanoparticles and free FSG nanoparticles in water (blue line) compared to FSG-*α* glucan nanoparticles (red line).

Fig. 3a shows the thermal profile of *α*-glucan nanoparticles in nanopure water containing 0.01 % sodium azide as an antimicrobial agent. The result of subtracting the background due to the presence of sodium azide showed that there was an exothermic process occurring within the *α*-glucan nanoparticles starting around 75 ºC and peaking around 102 ºC. Cooling the sample down and conducting a seccond scan revealed that there was little to no thermal activity, suggesting that the nanoparticles degraded completely around 110 ºC.

Fig. 3b shows that the thermal behavior of FSG nanoparticles over the same temperature range was surprisingly quite similar to *α*-glucan nanoparticles. An exothermic process was started around 70 ºC and completed by 102 ºC. A subsequent scan after cooling the sample back to 20 ºC revealed that FSG nanoparticles were also degraded; degradation was completed around 115 ºC.

The nano DSC thermal profile for FSG-*α* glucan combination nanoparticles is exhibited in Fig. 3c. It demonstrates that degradation was a two-step process. The first step in FSG-*α* glucan nanoparticle degradation began around 26 ºC and peaked at 66 ºC; this was larger contributor to heat loss during the overall degradation process. Considering that degradation for both FSG and *α* glucan nanoparticles did not start until 70–75 ºC, it is interpreted that the first step in FSG-*α* glucan nanoparticle degradation process was FSG being released from the *α* glucan nanoparticles. The second step of degradation began around 78 ºC, peaking at 87 ºC and was interpreted as degradation of both released FSG and any intact *α* glucan nanoparticles that remained. Neither steps were detected in a subsequent scan after cooling down, confirming that degradation was completed around 95 ºC during the first scan.

Comparison of thermal profiles between a 1:1 mixture of free FSG nanoparticles and free a-glucan nanoparticles was done to demonstrate that FSG-*α* glucan nanoparticles had distinctively different thermal properties than free FSG nanoparticles and free *α*-glucan nanoparticles. Surprisingly, there was a two-step thermal profile of the 1:1 mixture. The first step starts around 23 ºC, peaks around 50 ºC and ends near 65 ºC. Because this step peaks at nearly −2 mJ/s, which is small compared to the second step (∼−24 mJ/s), it is suspected that this first step is due to increased thermal mixing of the two separate nanoparticles. This thermal mixing was overshadowed by thermal degradation of the free nanoparticles which started around 70 ºC, peaked around 110 ºC and ended around 118 ºC.

Encapsulation rate of FSG within *α* glucan nanoparticles was approximated by comparing the ratio of the absorbance at 323 nm for FSG-*α* glucan nanoparticles versus that of FSG nanoparticles (Fig. S1). FSG nanoparticles were chosen because it is uniformly distributed in water after homogenization and does not form an oil-water layer; additionally, both samples have been put through nearly identical homogenization conditions. Data suggests that encapsulation rate is about 97.5 % of the homogenized FSG.

These findings are in comparison to encapsulation of other natural oils. Weng et al., used high-speed homogenization and electrospinning to nanoencapsulate camellia oil within zein fibers of 124 nm–282 nm diameter, resulting in 78 %–84 % encapsulation rate [29]. Liu et al., used protein micelles that were 50 nm–318 nm with a zeta potential range of −4 mV to −33 mV to encapsulate docosahexaenoic acid [30]. Lin et al., used poly-L-aspartic acid and chitosan to create 210 nm nanoparticles with ∼ −34 mV zeta potential to encapsulate *Litsea cubeba* essential oil at a nearly 70 % encapsulation rate [31]. Others have used soy lecithin based liposomes to encapsulate cannabis oil at an encapsulation rate of 91 % [32], fennel essential oil at a rate of 85 % [33] (references therein were able to encapsulate Artemisia annual essential oil, cardamom essential oil and garlic oil), *Ligusticum chuanxiong* Hort. essential oil at a rate of 80 % [34], and melon seed oil at an encapsulation rate up to 58 % [35].

## 4 Conclusions

α-Glucan nanoparticles encapsulating FSG formed 216 nm nanoparticles, which is larger than free *α*-glucan nanoparticles (125 nm in diameter) and larger than free FSG nanoparticles (190 nm in diameter). This size increase did not lead to an increase in polydispersity. The zeta potential of FSG-*α* nanoparticles was, on the other hand, in between that found for free FSG nanoparticles and free *α*-glucan nanoparticles. Thermal analysis showed that FSG-*α* glucan nanoparticles were thermally distinct from free FSG nanoparticles and free *α*-glucan nanoparticles, suggesting that FSG contents release can be induced by temperature. Future work will entail exploring maximizing the encapsulation rate of FSG within α glucan nanoparticles, characterizing release rates, and comparing encapsulation rates of systems, like soy lecithin liposomes.

## Declaration of Competing Interest

The authors declare that they have no known competing financial interests or personal relationships that could have appeared to influence the work reported in this paper.

## Data Availability

Data will be made available on request.

## Appendix Supplementary materials

Image, application 1
