# Supplementary material for: Biophysical characterization of α-glucan nanoparticles encapsulating feruloylated soy glycerides (FSG)
Source: Biotechnol Rep (Amst). 2023 Nov 2;40:e00817. doi: 10.1016/j.btre.2023.e00817 (PMC10658199; doi:10.1016/j.btre.2023.e00817)
Supplement: Supplementary file 1 [file mmc1.docx]

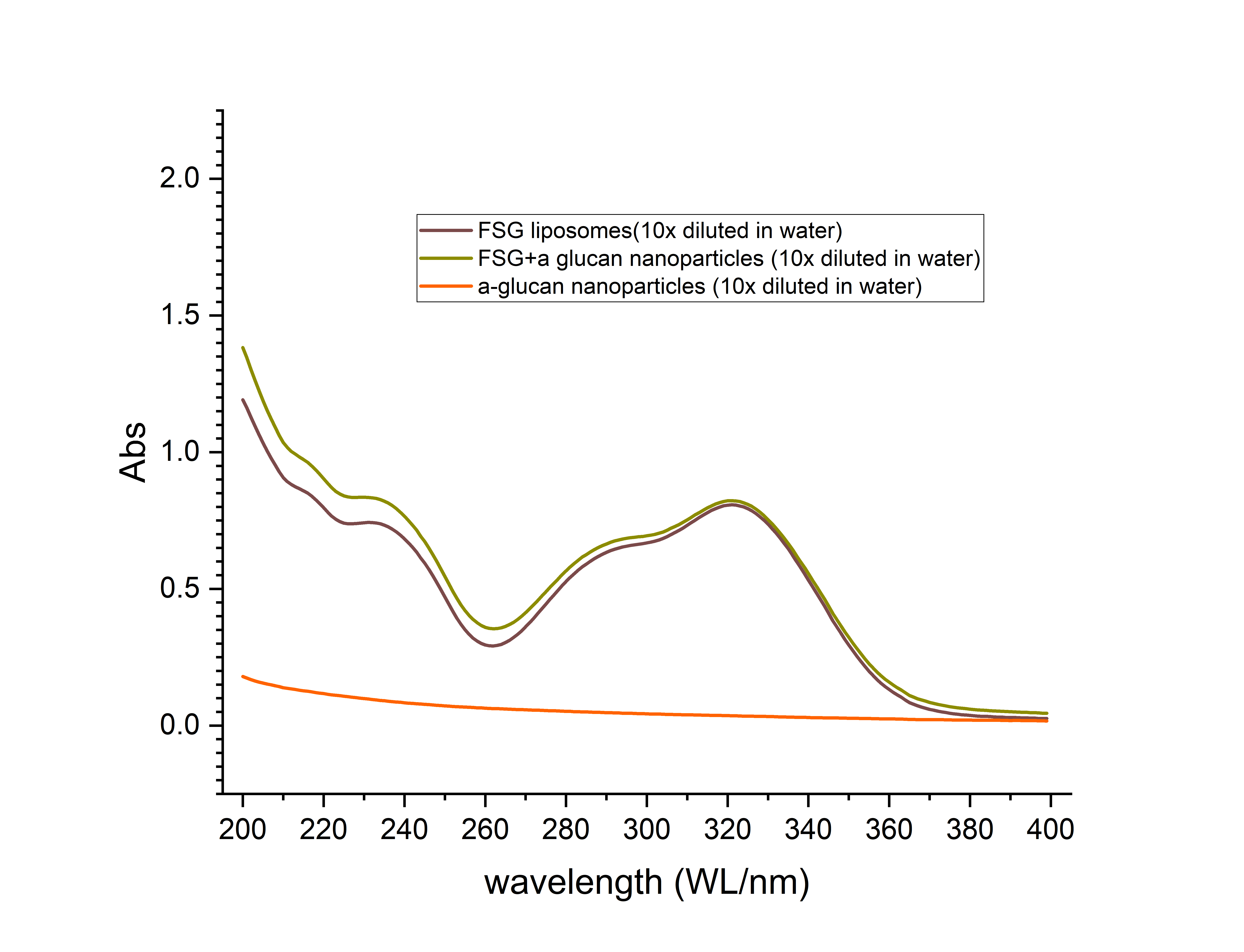


Figure S1. Absorbance spectra of FSG homogenized into liposomes, homogenized alpha-glucan nanoparticles, and FSG/α glucan nanoparticles.
